# Prolonged coma due to amitriptyline overdose and genetic polymorphism: a case report

**DOI:** 10.1186/s13256-022-03294-x

**Published:** 2022-03-16

**Authors:** Tijs van de Wint, Aurelia H. M. de Vries Schultink, Arend Jan Meinders, Ankie Harmsze, Peter Bruins

**Affiliations:** 1 Department of Anesthesiology, Intensive Care and Pain Management, St. Antonius Hospital, Amsterdam, The Netherlands; 2 Department of Clinical Pharmacy, St. Antonius Hospital, Nieuwegein, The Netherlands; 3 Department of Internal Medicine and Intensive Care, St. Antonius Hospital, Nieuwegein, The Netherlands

**Keywords:** Case report, Coma, Intoxication, Tricyclic antidepressant, Amitriptyline, Metabolizer status

## Abstract

**Background:**

Reduced consciousness has a wide variety of possible causes, not infrequently being toxic in nature. An intoxication might be obvious, but in this paper an unexpected case with a tricyclic antidepressant is presented.

**Case presentation:**

A 76-year-old caucasian female was found unconscious. Primary diagnostic evaluation, including a negative drugs of abuse test, did not give direction to any clear cause. Yet an intraventricular conductive disorder with widening of the QRS complex and electroencephalogram abnormalities did suggest a possible drug effect. Heteroanamnestic information led to the suspicion of an amitriptyline intoxication, which was confirmed by further laboratory analysis. The patient remained comatose for several days. High concentrations of amitriptyline indicated a large overdose of amitriptyline and, in combination with a cytochrome P450 2D6 poor metabolizer status, could explain the long persistence of her comatose state.

**Conclusion:**

We present a tricyclic antidepressant intoxication, where the patient is thought to have taken a large amount of amitriptyline at once, which, in combination with a cytochrome P450 2D6 poor metabolizer status, led to an unusual long persistence of her coma.

## Introduction

Reduced consciousness and coma have a wide range of possible causes, from primary neurologic, metabolic to cardiopulmonary dysfunction [1]. One of the more common causes being an intoxication. Sometimes an intoxication is obvious or deliberate, but this case represents an example of a genetic variation in cytochrome P450 2D6 (CYP2D6) in persistent coma.

## Case presentation

A 76-year-old caucasian female was presented to the emergency department unconscious and with possible aspiration with some vomit in the oropharynx. The differential diagnosis included postanoxic encephalopathy, a metabolic disorder, infectious cause, nonconvulsive status epilepticus, intoxication, meningitis, and conversion.

The patient looked neglected and underweight. Vital signs showed a heart rate of 105/minute, blood pressure 150/80 mmHg, respiratory rate 21/minute, SpO_2_ 98% with nonrebreathing mask, and temperature 36.9 °C. The patient was unresponsive, with a Glasgow Coma scale of 3, pupils were 6 mm in diameter, equal and reactive to light. She was immediately intubated for airway protection and mechanically ventilated. Fiberoptic bronchoscopy was negative for signs of aspiration and pressures were within normal range.

**Fig. 1:**
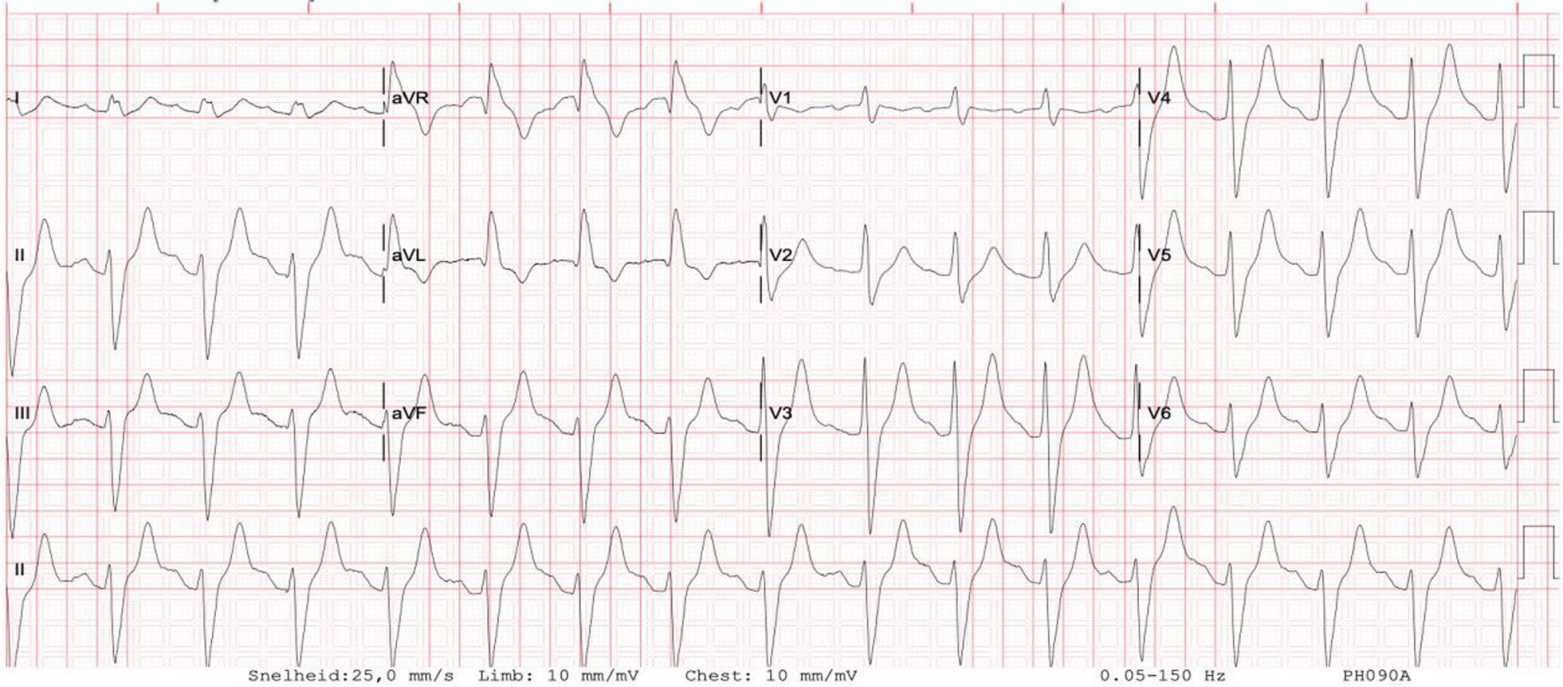
The electrocardiogram on admission showed an intraventricular conductive disorder with QRS of 150 ms

Laboratory results: hemoglobin (Hb) 7.0 mmol/L, platelets 151 × 10 ^9^/L, white blood cells (WBC) 6.5 × 10 ^9^/L, creatinine 91 μmol/L, urea 12.5 mmol/L, C-reactive protein (CRP) 114 mg/L, Na 138 mmol/L, K 3.4 mmol/L, Ca 2.24 mmol/L, Mg 0.84 mmol/L, creatine kinase (CK) 20 U/L, aspartate aminotransferase (AST) 17 U/L, alanine aminotransferase (ALT) 15 U/L, alkaline phosphatase 51 U/L, albumin 33.5 g/L, ammonia 11 mmol/L, glucose 9 mmol/L, arterial blood gas pH 7.30, pO_2_ 9.44 kPa, pCO_2_ 6.72 kPa, HCO_3_ 24.1 mmol/L, and base excess 2.7 mmol/L. Initial electrocardiogram (ECG) showed an intraventricular conductive disorder (Fig. 1).

Qualitative analyses for drugs of abuse (Syva RapidTest d.a.u., Siemens Healthcare Diagnostics Ltd., Frimley, Camberley, UK) in the urine were negative for cannabis, opiates, (methyl)amphetamines, methadone, benzodiazepines, and cocaine. Paracetamol plasma concentration was < 10 mg/L. A computed tomography (CT) angiography of the head showed no signs of recent ischemia nor significant intracranial vessel occlusions.

**Fig. 2:**
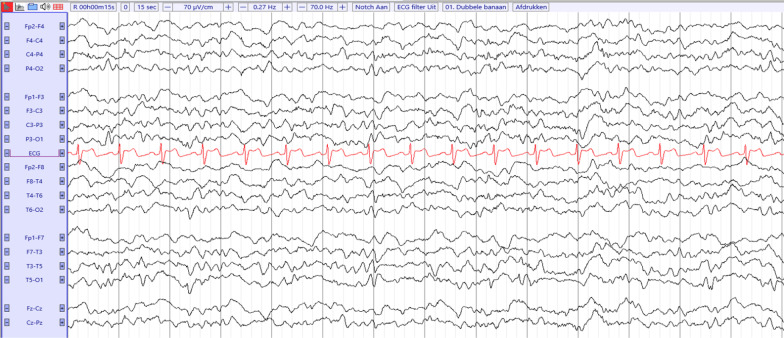
Electroencephalogram on admission. Symmetrical pattern with excess of slow activity with generalized predominant synchronous frequencies of theta (4–7 Hz) and delta (1–3 Hz) throughout the background, most likely appropriate to the diagnosis of metabolic or toxic encephalopathy

She was transferred to the intensive care unit (ICU). An electroencephalogram (EEG) showed diffuse nonspecific nonepileptiform background slowing (Fig. 2). This is commonly observed in patients with an encephalopathy or induced by a drug intoxication [2].

The next morning, the patient’s family clarified that the patient had complaints of anxiety and depressive thoughts, suffered from significant weight loss, and had a poor performance status. She had gone to bed at around midnight in fair condition and was found comatose by her daughter next morning at 11:00 AM. There were no signs of a deliberate overdose, for example no empty medicine strips were found. Contact with her general practitioner revealed that amitriptyline had been prescribed intermittently from 2014 to September 2019. However, amitriptyline had not been prescribed in the year prior to her admission. Therefore, the patient was believed to have resumed medication on her own initiative.

**Fig. 3:**
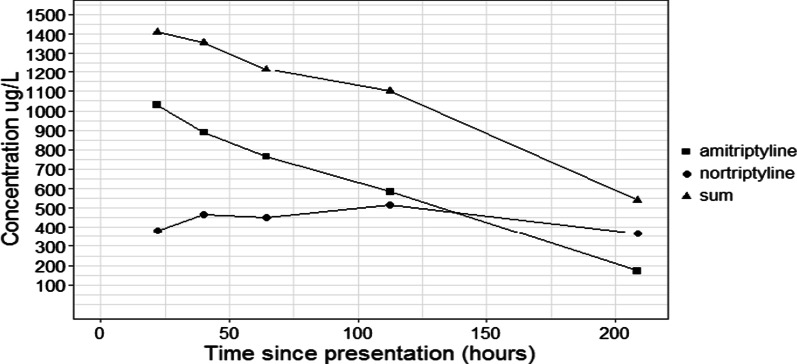
Plasma concentrations of amitriptyline and active metabolite nortriptyline

This would also explain her ECG changes, mydriasis due to anticholinergic effects, and her comatose state. Because of the suspicion of a tricyclic antidepressant (TCA) intoxication, plasma levels were determined. An amitriptyline plasma concentration of 1030 μg/L and a nortriptyline (active metabolite) plasma concentration of 380 μg/L were found, resulting in a sum concentration of 1410 μg/L (therapeutic range sum 100–300 μg/L) (Fig. 3). Plasma concentrations of amitriptyline and nortriptyline higher than 500 μg/L are considered to be potentially toxic.

Sodium bicarbonate was administered to lower the free fraction of amitriptyline [3]. In addition, symptomatic treatment was initiated, using diazepam for convulsions and extended cardiac monitoring. Since concentrations were high and comatose state persisted, impaired clearance was suspected. Analysis of genetic variations in cytochrome (CYP)450 isoenzymes, which play a major role in metabolism of amitriptyline, was performed. Genetic variations in CYP2C19 and CYP2D6 were determined by real-time PCR (validated TaqMan StepOnePlus assay). DNA sequence analysis was used to validate the genotyping procedure. The patient was found to be a CYP2D6 poor metabolizer (homozygous CYP2D6*4 and homozygous CYP2D6*10) and a CYP2C19 extensive metabolizer (CYP2C19*1/*17). CYP2D6 poor metabolizer status leads to reduced degradation of nortriptyline, increasing the concentration of this metabolite.

**Fig. 4:**
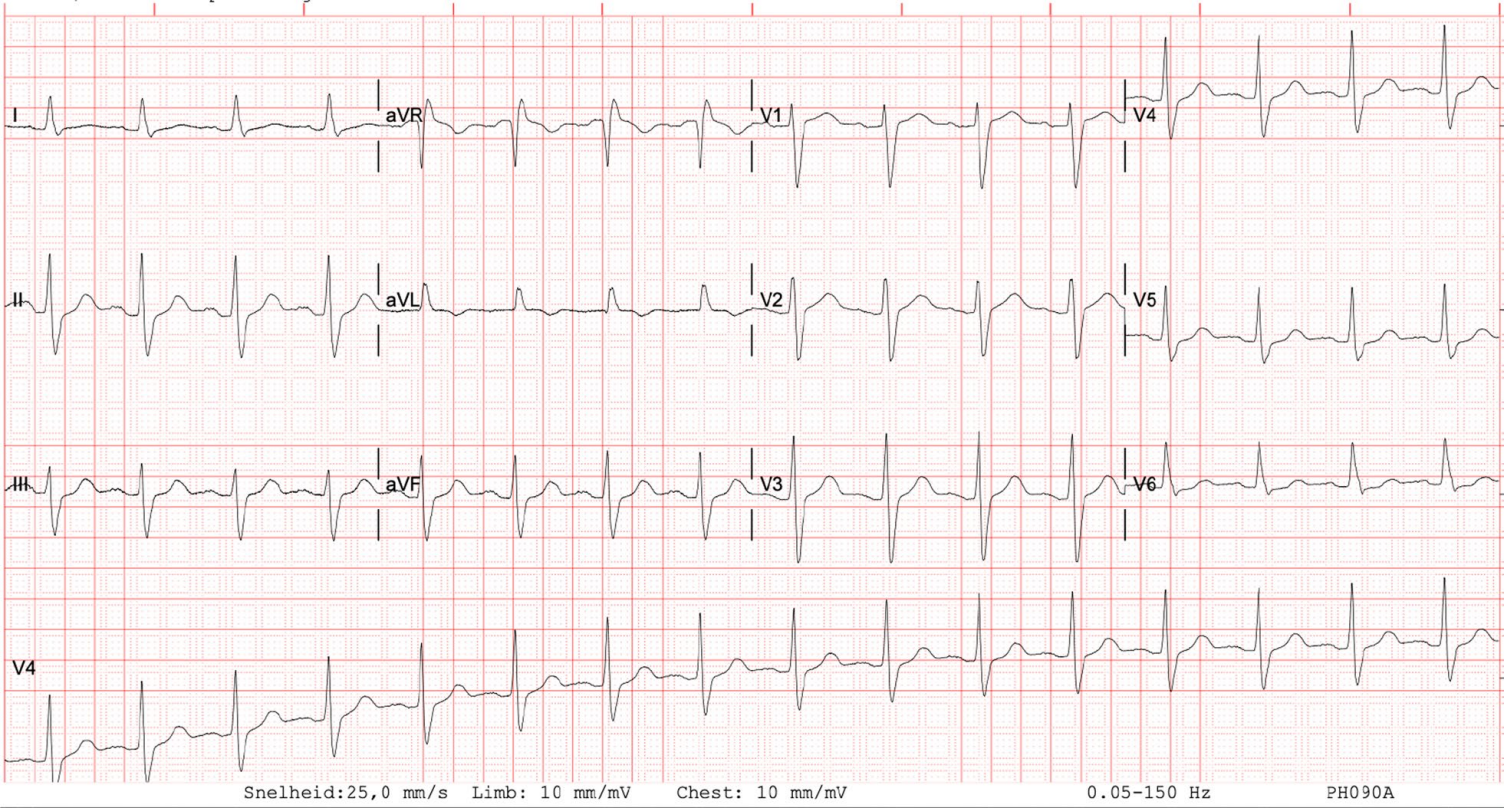
Electrocardiogram 3 days after admission. Normalization of intraventricular conductance and QRS duration

Three days after admission, her Glasgow Coma score (GCS) started to improve to an E2M4Vt and was back to normal on day 7. ECG changes gradually resolved (Fig. 4). She was extubated on day 5. Psychiatric evaluation showed no signs of a deliberate overdose or attempted suicide. She was discharged in a reasonably good condition 10 days after admission.

## Discussion and conclusions

The approach of an unconscious patient can be challenging as various causes may play a role in the situation. The differential diagnosis of the nontraumatic unconscious patient may be divided into four categories, that is neurological, metabolic, diffuse physiological brain dysfunction, and psychiatric or functional [1].

In our patient, coma was clinically evident and persistent. The laboratory results did not give direction to any possible cause, that is, summarizing the results showed an isolated elevation of C-reactive protein, without any other sign of inflammation, no electrolyte disorders, mild increase of plasma creatinine, and normal liver function tests. Drugs of abuse (DOA)-test did not reveal any possible cause of drug abuse. Both the intraventricular conductive disorder with widening of the QRS complex and the diffuse nonspecific nonepileptiform symmetrical slowing with generalized delta activity on the EEG suggested a possible drug effect, and in combination with important heteroanamnestic information, led to the suspicion of an amitriptyline intoxication. The mydriasis due to anticholinergic effects combined with the myocardial conductive changes further supported this possible diagnosis. Previous studies showed that QRS prolongation above 100 ms, as in our patient with 150 ms, also supported the idea that this was a very serious intoxication [4].

Moreover, we observed very high concentrations of amitriptyline and nortriptyline. Amitriptyline and its active metabolite nortriptyline are both TCAs, and their mechanism of action relies on reducing the reuptake of, respectively, serotonin and noradrenaline in the synapses of the central nervous system.

Amitriptyline is predominantly metabolized by CYP2C19 into its active metabolite nortriptyline. Both amitriptyline and nortriptyline are metabolized by CYP2D6 into hydroxy metabolites that are less active than amitriptyline, and into inactive metabolites [5]. Typically, the half-life of amitriptyline is 12–25 hours and nortriptyline is 36 hours [6]. Since both amitriptyline and nortriptyline are metabolized by CYP2D6, CYP2D6 poor metabolizer status can increase concentrations of both amitriptyline and nortriptyline. However, nortriptyline is more dependent on CYP2D6 metabolism compared with amitriptyline, which depends more on CYP2C19. Moreover, CYP2D6 poor metabolizer status can increase the half-life of nortriptyline to 100 hours, whereas its effect on amitriptyline half-life is much lower [7] In this case, the amitriptyline and nortriptyline half-lives were approximated (based on the last two concentration time points) to be prolonged to 54 and 195 hours, respectively.

The CYP2D6 poor metabolizer status alone is not able to explain the comatose state of the patient described in this case report. Firstly, the CYP2D6 polymorphism can explain the high (and increasing) concentrations of nortriptyline, but not the increased concentration of amitriptyline, since it is less dependent on CYP2D6 for metabolism. Secondly, the patient’s prescription for amitriptyline was a daily dose of 25 mg for sleep augmentation, a sixfold lower dose compared with the 150 mg daily recommended for depression. According to the Clinical Pharmacogenetics Implementation Consortium (CPIC) guidelines, TCAs should not be prescribed to CYP2D6 poor metabolizer patients, or, if warranted, the dose should be decreased to 50% under monitoring of plasma concentrations and side effects. However, for the indication of neuropathic pain, no recommendations are made for using a dose of 25 mg daily, except for side effect monitoring, since it is expected that at this dose, no clinically relevant increase of plasma concentrations of amitriptyline and nortriptyline occur [8].

Therefore, the patient described in this case report is expected to have taken a larger amount of amitriptyline at once opposed to the theory of accumulation of therapeutic dose via delayed degradation of nortriptyline. High concentrations of amitriptyline indicate great overdose of amitriptyline and, in combination with CYP2D6 poor metabolizer status, they may explain the long persistence of comatose status of this patient.

## Data Availability

All data generated or analyzed during this study are available from the corresponding author upon reasonable request.
